# Supplementary material for: Vegetation dieback in the Mississippi River Delta triggered by acute drought and chronic relative sea-level rise
Source: Nat Commun. 2024 Apr 25;15:3518. doi: 10.1038/s41467-024-47828-x (PMC11045820; doi:10.1038/s41467-024-47828-x)
Supplement: Supplementary file 1 — Supplementary Information [file 41467_2024_47828_MOESM1_ESM.pdf]

Vegetation Die-off in the Mississippi River Delta Triggered by Acute Drought and Chronic Relative Sea-level Rise

T. Elsey-Quirk*, A. Lynn, M. D. Jacobs, R. Diaz, J. Cronin, L. Wang, H. Huang, D. Justic

*Corresponding author. Email: tquirk@lsu.edu

This file includes:

Supplementary Figures 1 to 15

Supplemental Information

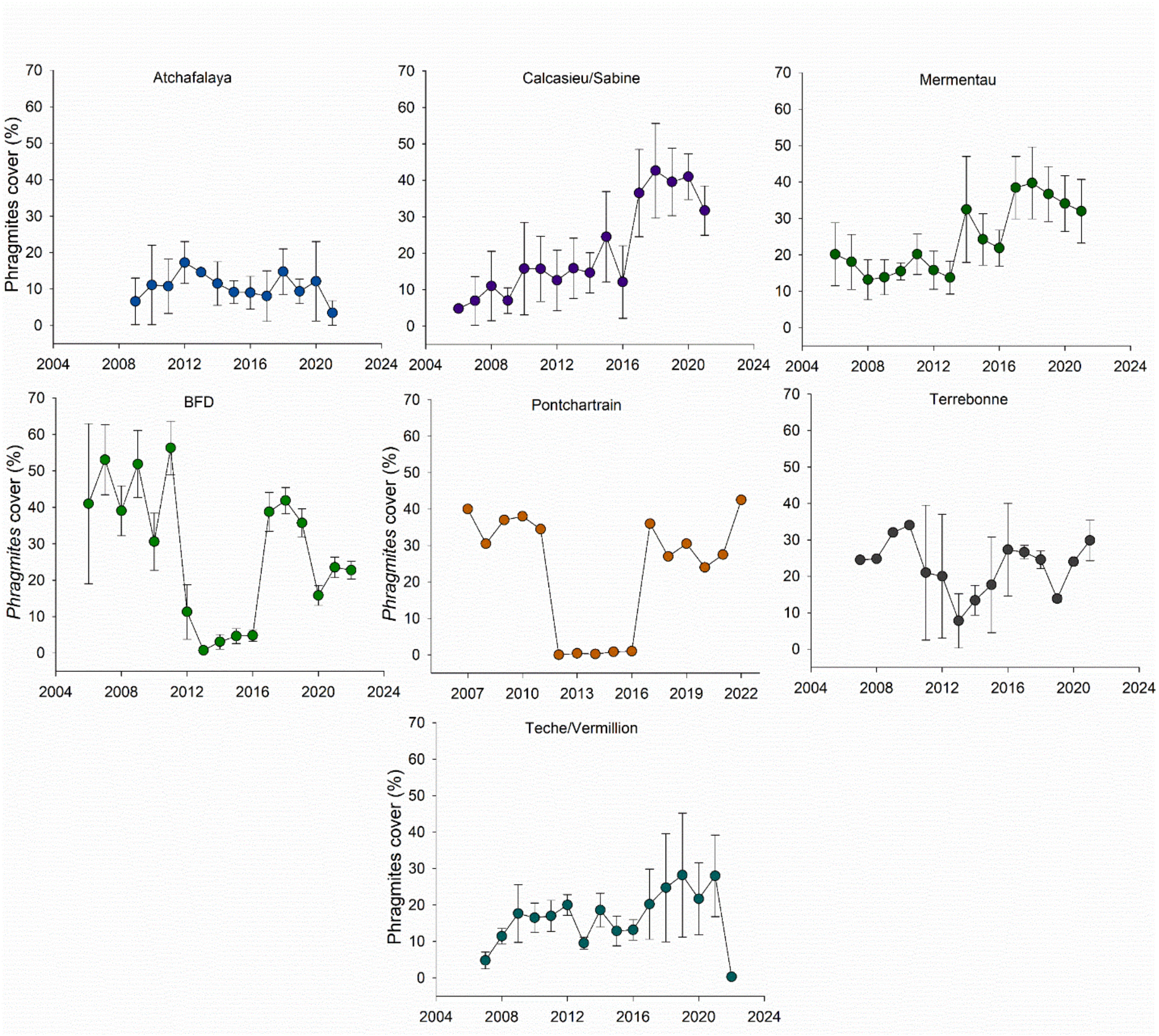

Supplementary Figure 1. ***Phragmites australis*** **cover dynamics 2007 - 2022 in the seven hydrologic basins of the Mississippi River Deltaic and Chenier Plain regions of Louisiana at Coastwide Reference Monitoring System stations.** From left to right, top panels: Atchafalaya, Calcasieu/Sabine, and Mermentau basins, middle panels: Birdfoot delta (BFD), Pontchartrain, and Terrebonne basins, and bottom panel: Teche/Vermillion basin.

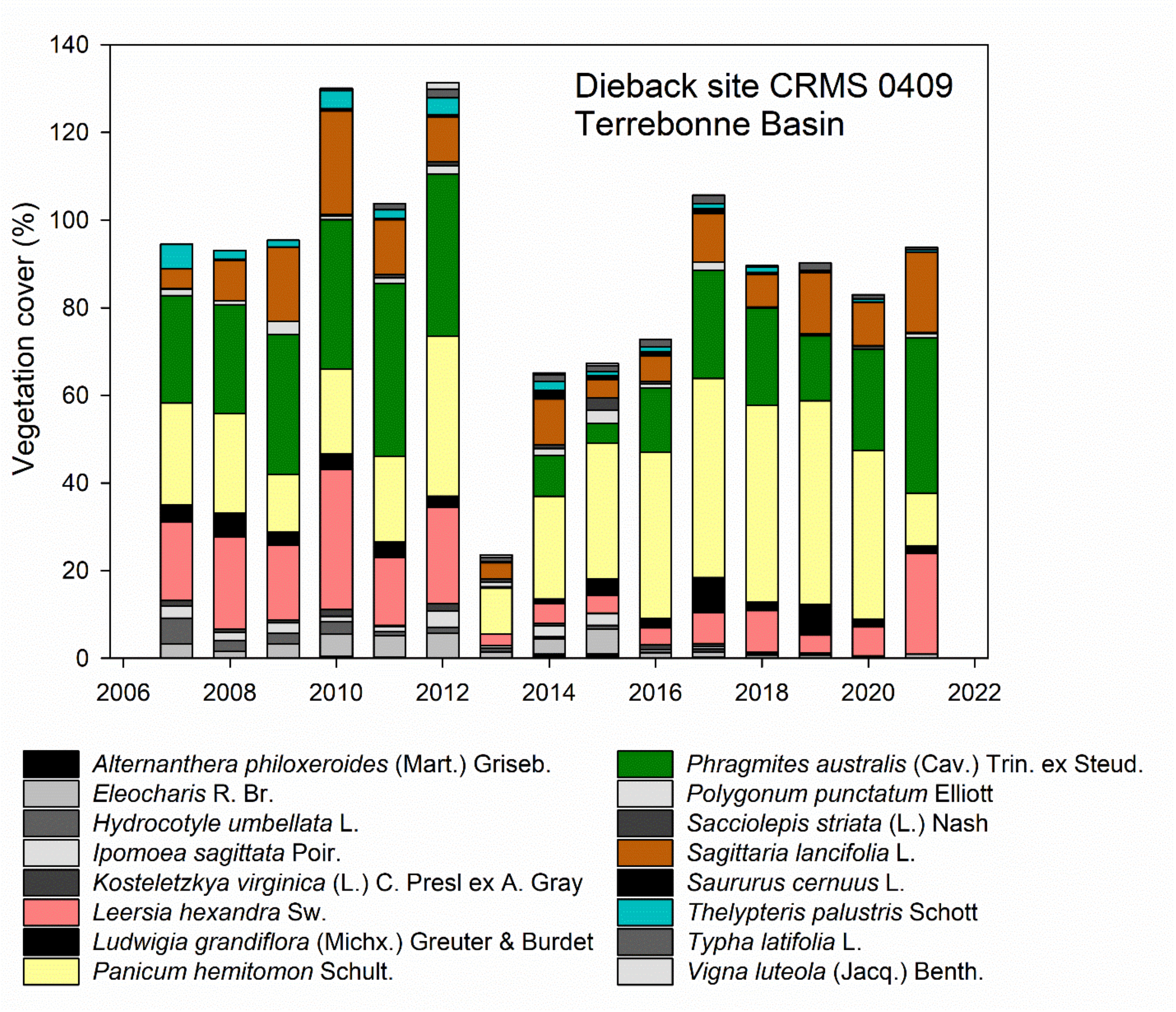

Supplementary Figure 2. Vegetation cover dynamics 2007 - 2021 at a dieback area (CRMS site 0409) in the Terrebonne Basin, Mississippi River Delta, Louisiana. Data show dieback in 2013, recovery 2014 – 2016 to pre-dieback conditions by 2017.

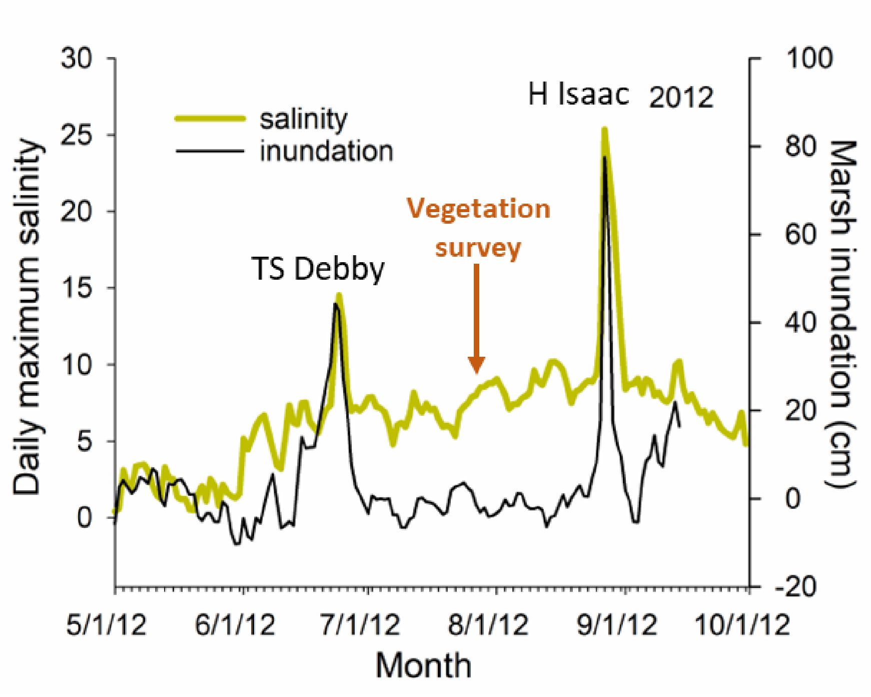

Supplementary Figure 3. Timing of vegetation survey when die-off was first documented in summer 2012.

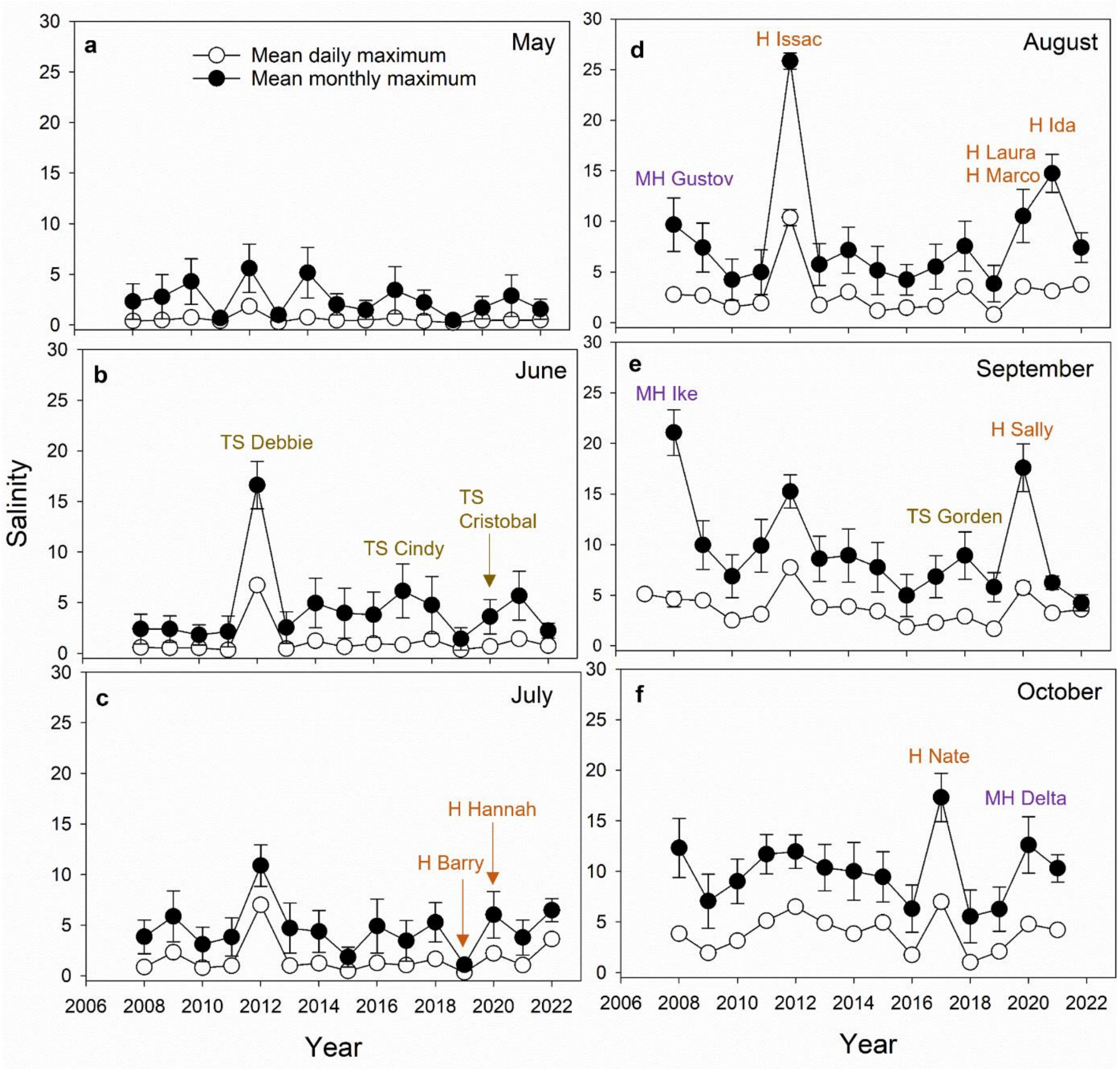

Supplementary Figure 4. Mean and maximum salinity May – October in the die-off marshes, 2008 – 2021. a – f. May – October, respectively.

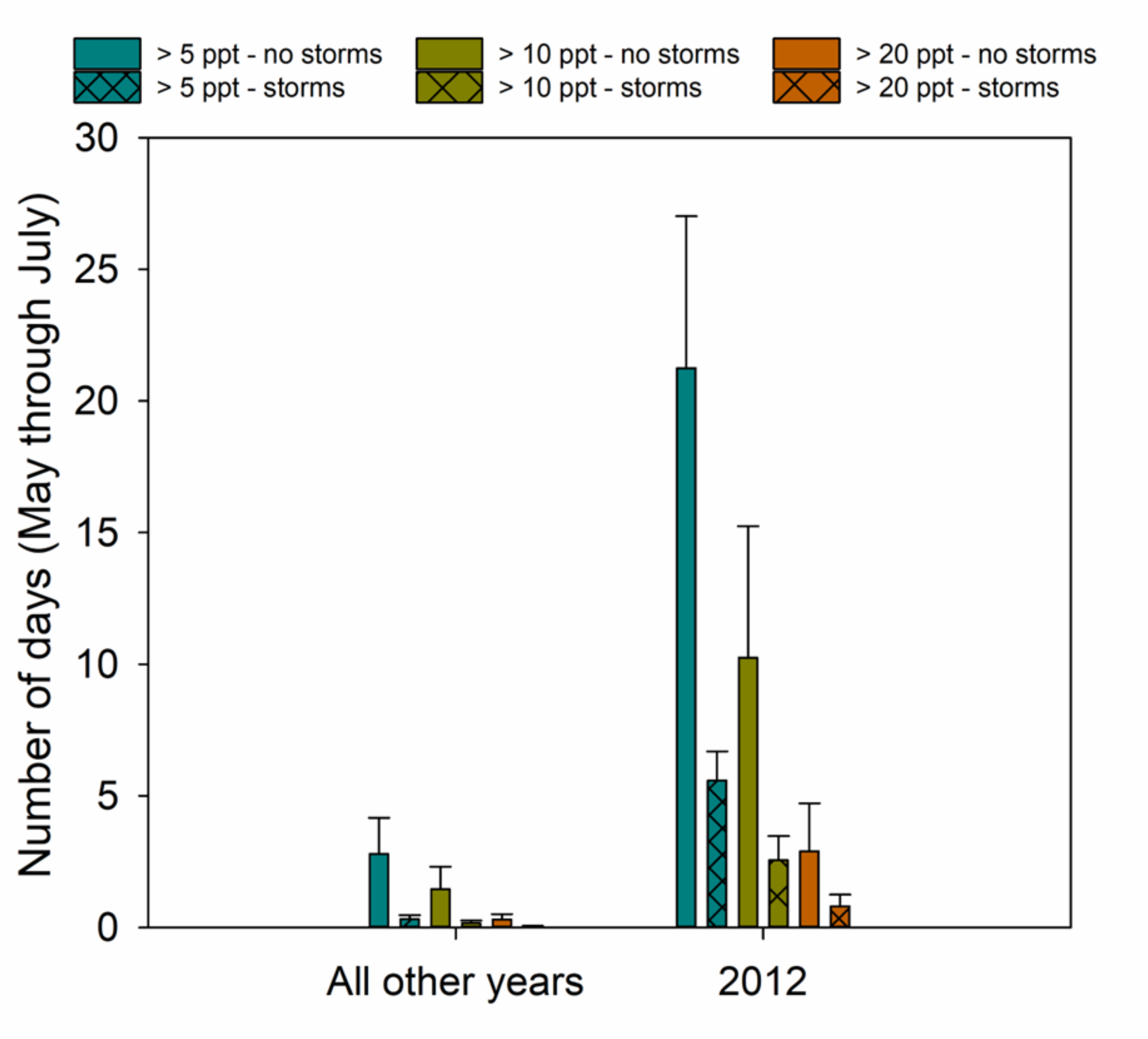

Supplementary Figure 5. Number of days salinity exceeded 5, 10 and 20 psu during non-cyclone and cyclone periods May – July. Values are averages across years 2008 – 2022 and 2012 in dieback Coastwide Reference Monitoring System stations (n = 15).

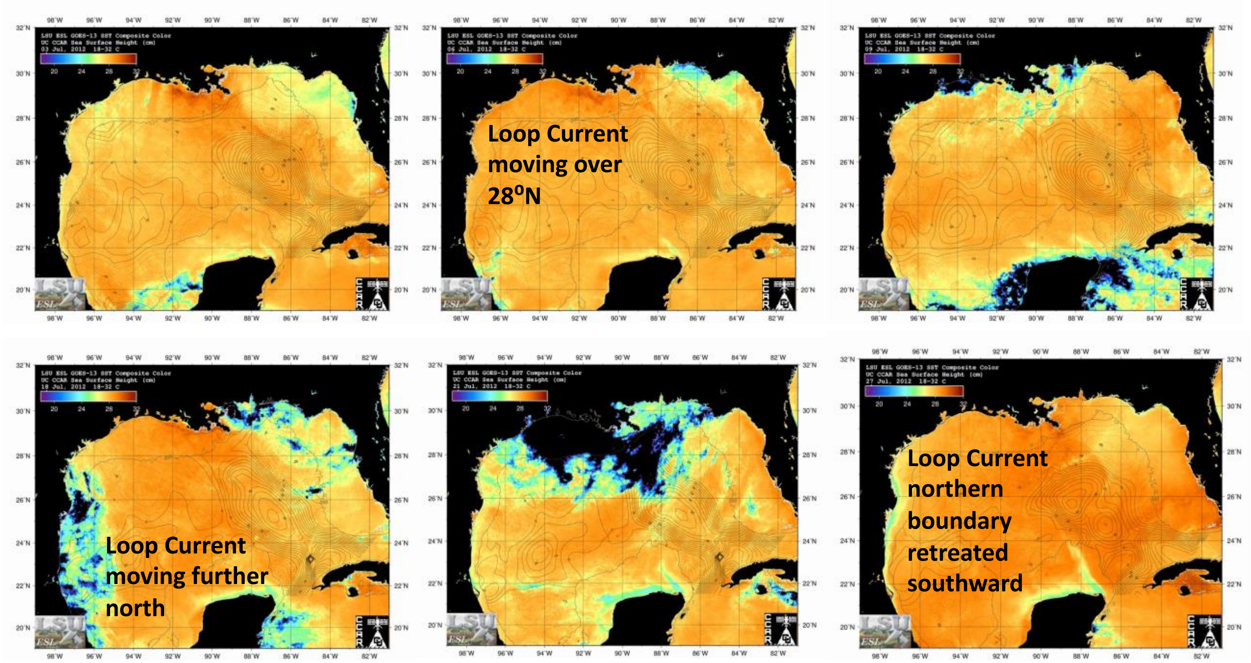

Supplementary Figure 6. Timeseries of showing northern incursion of loop current in the Gulf of Mexico in July 2012. From left to right, top panels are from dates July 3, 6, and 9 and bottom panels are from dates July 18, 21, and 27, 2012.

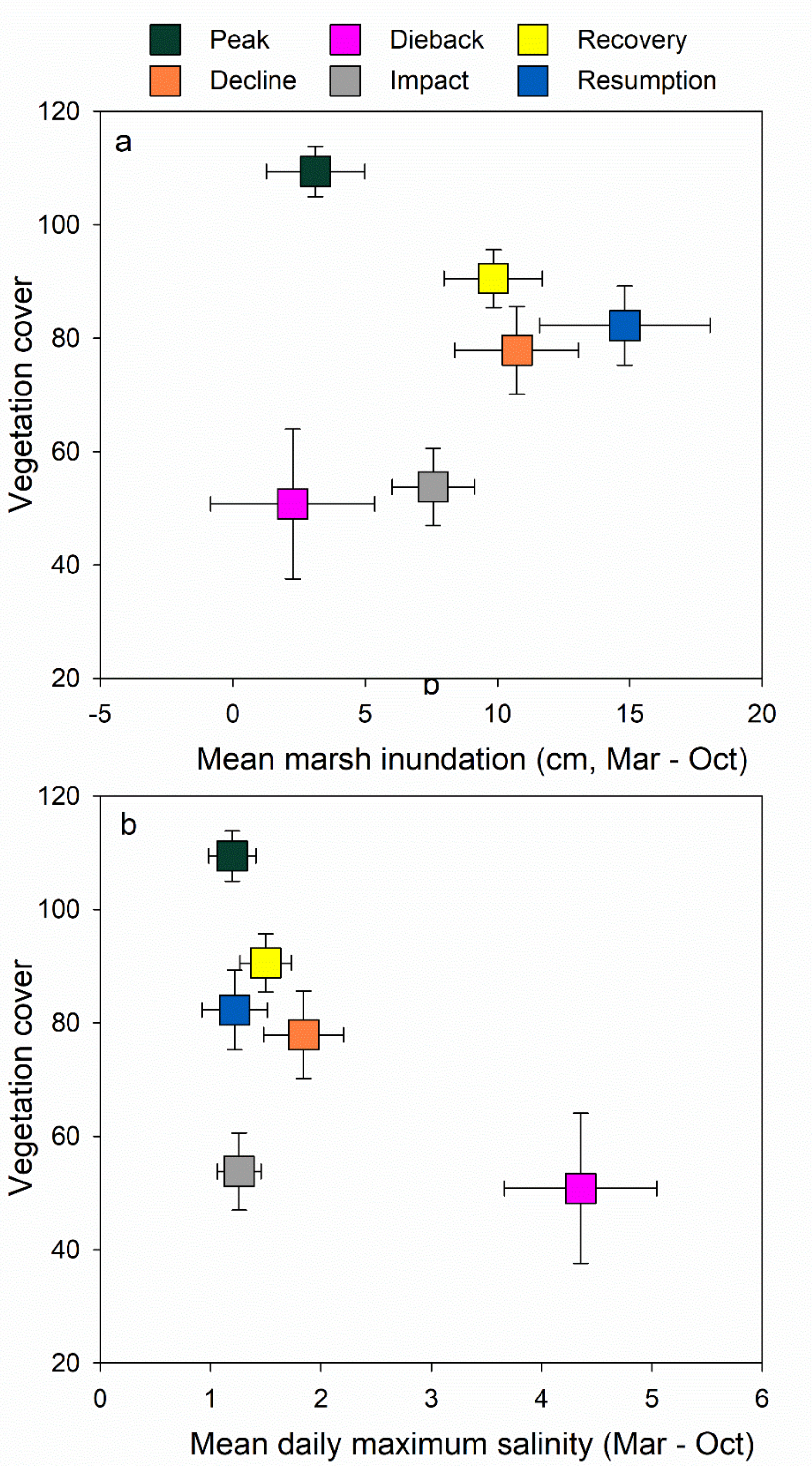

Supplementary Figure 7. Relationship between vegetation cover and mean marsh inundation depth (a) and mean daily maximum salinity (b) for different vegetation states.

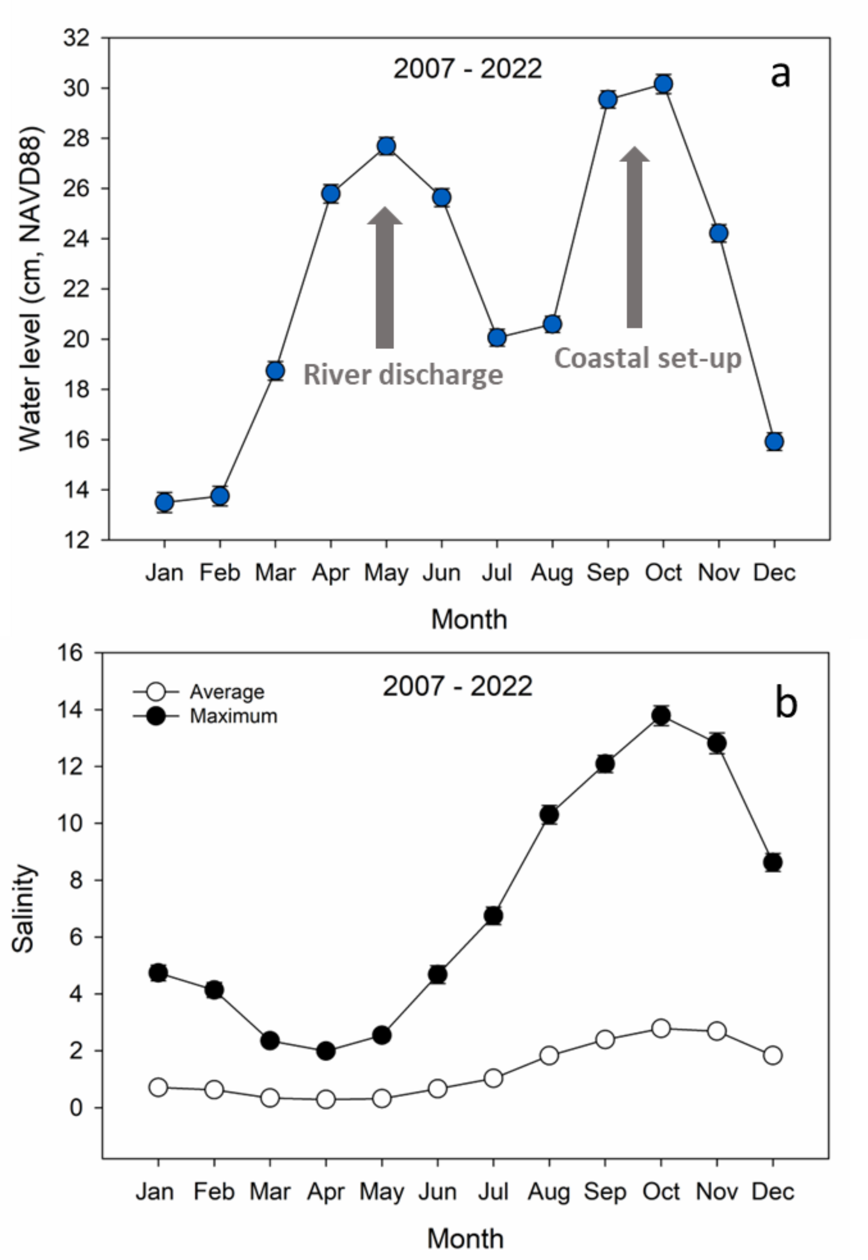

Supplementary Figure 8. Seasonal water level and salinity across the Birdfoot Delta. a. mean month water level and b mean and maximum monthly salinity from 2007 – 2022. Data are monthly means averaged over 16-years across 14 Coastwide Reference Monitoring stations (n = 14 ± standard error).

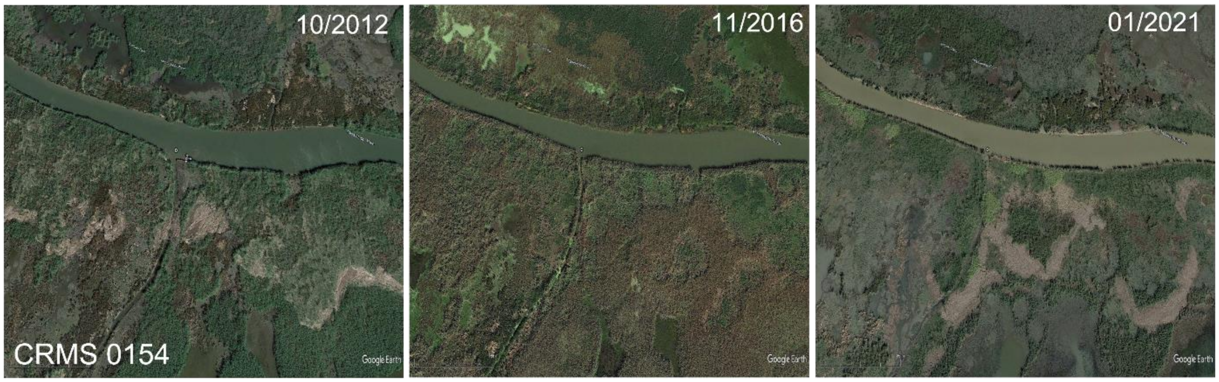

Supplementary Figure 9. Satellite images of Coastwide Reference Monitoring System (CRMS) station 0154 in the Birdfoot Delta. Google Earth, 2023. From left to right imagery is from October 2012, November 2016, and January 2021.

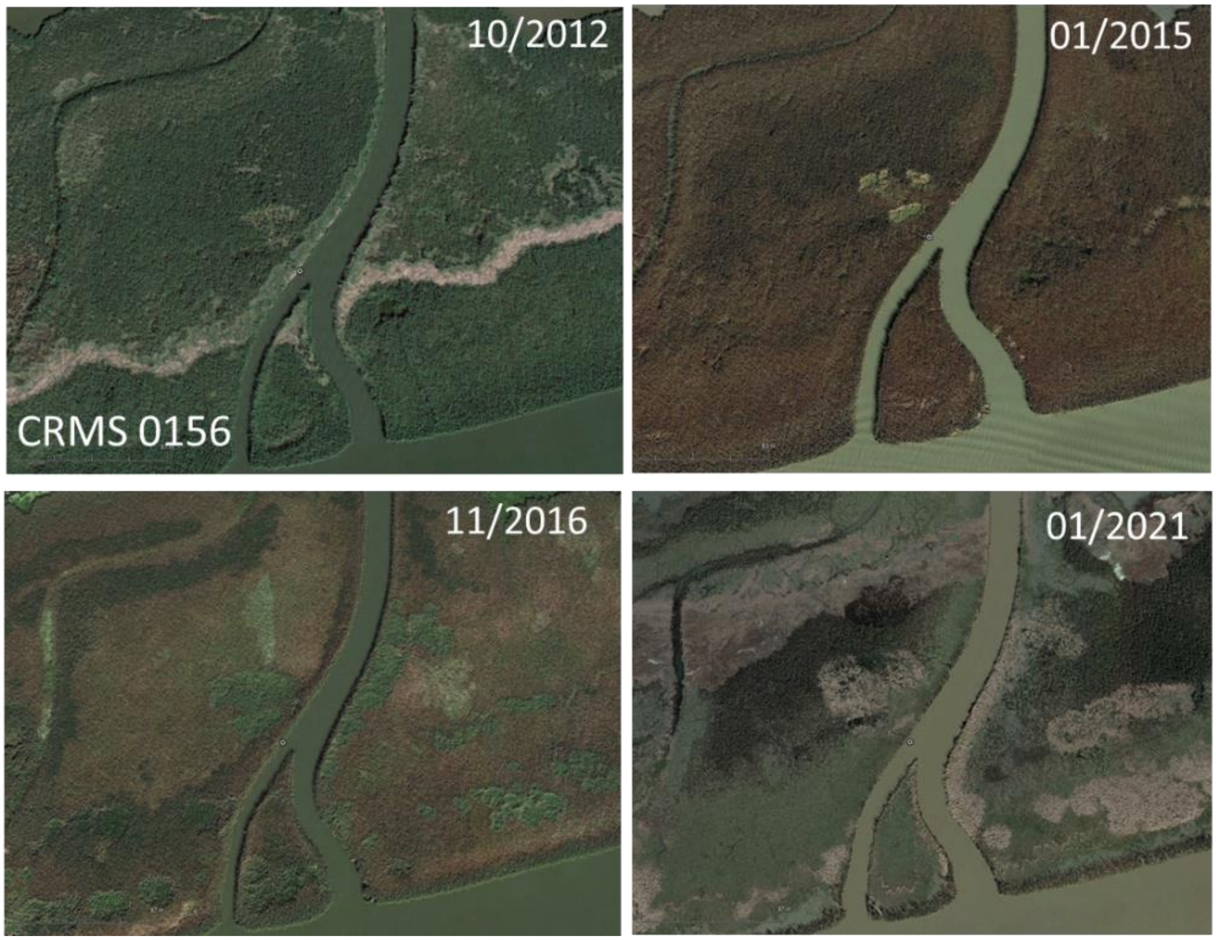

Supplementary Figure 10. Satellite images of Coastwide Reference Monitoring System (CRMS) station 0156 in the Birdfoot Delta. Google Earth, 2023. From left to right top panels, are imagery from October 2012 and January 2015, and bottom panel from November 2016 and January 2021.

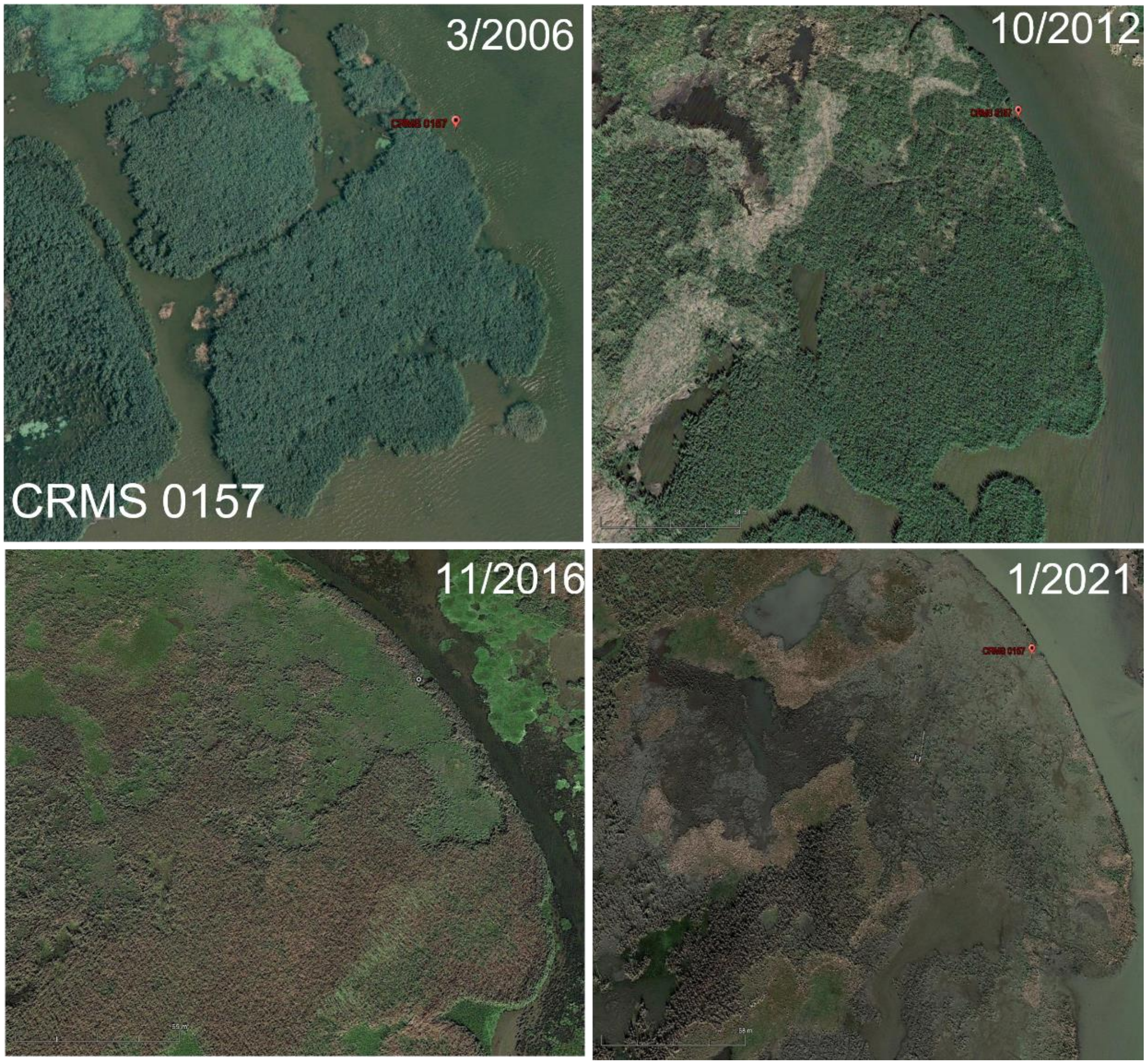

Supplementary Figure 11. Satellite images of Coastwide Reference Monitoring System (CRMS) station 0157 in the Birdfoot Delta. Google Earth, 2023. From left to right top panels, are imagery from March 2006 and October 2012, and bottom panel from November 2016 and January 2021.

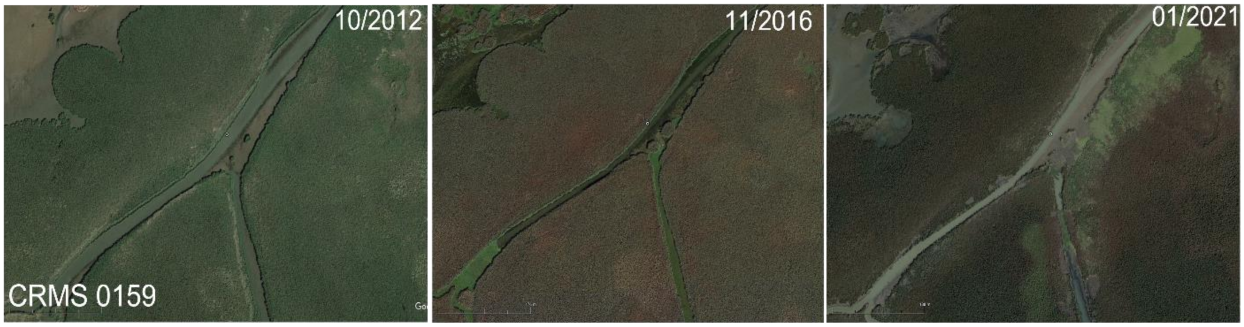

Supplementary Figure 12. Satellite images of Coastwide Reference Monitoring System (CRMS) station 0159 in the Birdfoot Delta. Google Earth, 2023. From left to right imagery is from October 2012, November 2016, and January 2021.

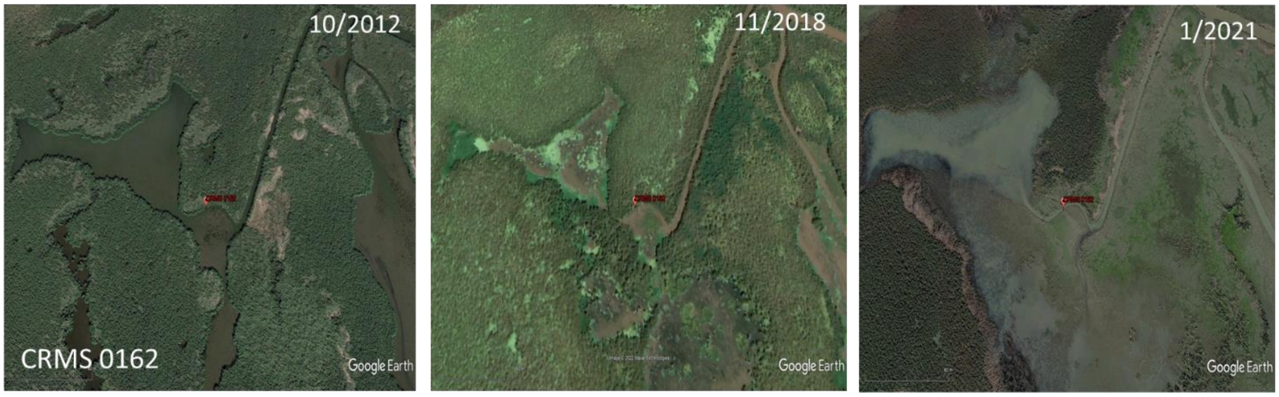

Supplementary Figure 13. Satellite images of Coastwide Reference Monitoring System (CRMS) station 0162 in the Birdfoot Delta. Google Earth, 2023. From left to right imagery is from October 2012, November 2018, and January 2021.

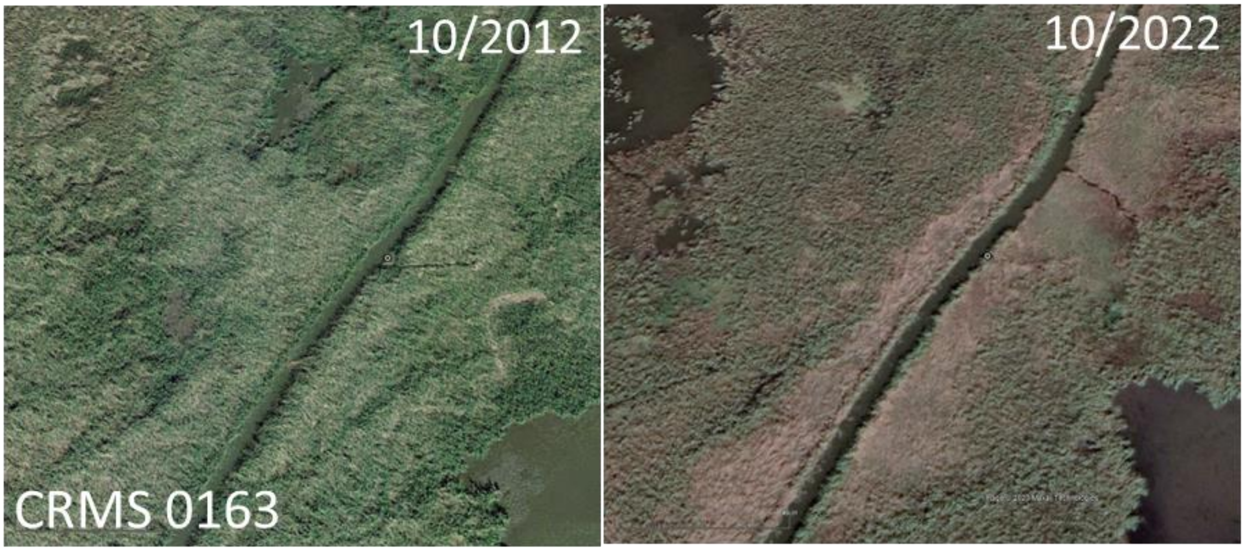

Supplementary Figure 14. Satellite images of Coastwide Reference Monitoring System (CRMS) station 0163 in the Birdfoot Delta. Google Earth, 2023. From left to right imagery is from October 2012 and October 2022.

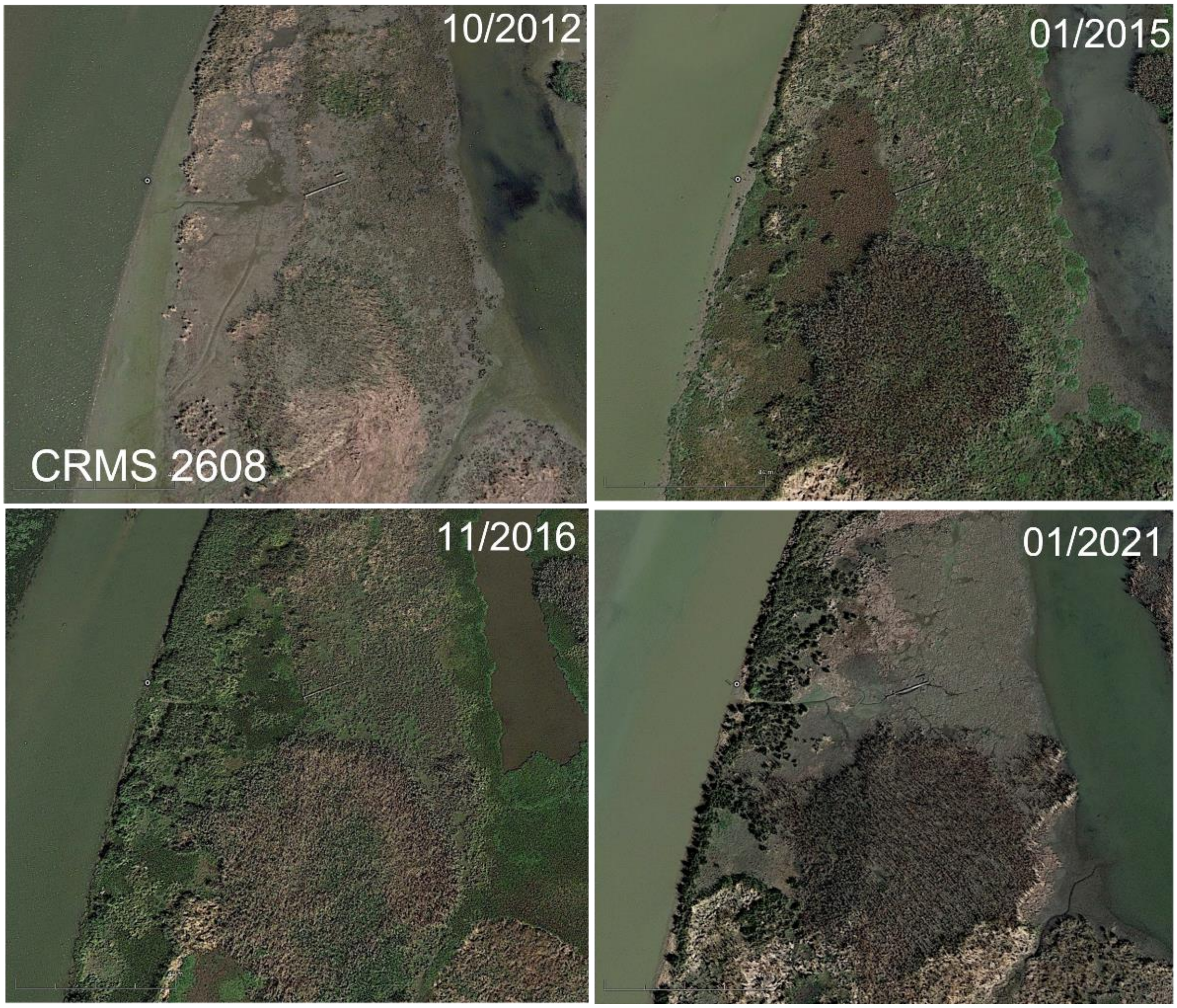

Supplementary Figure 15. Satellite images of Coastwide Reference Monitoring System (CRMS) station 2608 in the Birdfoot Delta. Google Earth, 2023. From left to right top panels, are imagery from October 2012 and January 2015, and bottom panel from November 2016 and January 2021.
