# Supplementary material for: Vegetation dieback in the Mississippi River Delta triggered by acute drought and chronic relative sea-level rise
Source: Nat Commun. 2024 Apr 25;15:3518. doi: 10.1038/s41467-024-47828-x (PMC11045820; doi:10.1038/s41467-024-47828-x)
Supplement: Supplementary file 2 — Reporting Summary [file 41467_2024_47828_MOESM2_ESM.pdf]

nature portfolio

Corresponding author(s): Tracy Elsey-Quirk
Last updated by author(s): 2024/03/28

Reporting Summary

Nature Portfolio wishes to improve the reproducibility of the work that we publish. This form provides structure for consistency and transparency in reporting. For further information on Nature Portfolio policies, see our Editorial Policies and the Editorial Policy Checklist.

Statistics

For all statistical analyses, confirm that the following items are present in the figure legend, table legend, main text, or Methods section.

n/a	Confirmed	
☐	☒	The exact sample size (*n*) for each experimental group/condition, given as a discrete number and unit of measurement
☐	☒	A statement on whether measurements were taken from distinct samples or whether the same sample was measured repeatedly
☐	☒	The statistical test(s) used AND whether they are one- or two-sided<br>*Only common tests should be described solely by name; describe more complex techniques in the Methods section.*
☐	☒	A description of all covariates tested
☐	☒	A description of any assumptions or corrections, such as tests of normality and adjustment for multiple comparisons
☐	☒	A full description of the statistical parameters including central tendency (e.g. means) or other basic estimates (e.g. regression coefficient) AND variation (e.g. standard deviation) or associated estimates of uncertainty (e.g. confidence intervals)
☐	☒	For null hypothesis testing, the test statistic (e.g. *F*, *t*, *r*) with confidence intervals, effect sizes, degrees of freedom and *P* value noted<br>*Give P values as exact values whenever suitable.*
☐	☒	For Bayesian analysis, information on the choice of priors and Markov chain Monte Carlo settings
☐	☒	For hierarchical and complex designs, identification of the appropriate level for tests and full reporting of outcomes
☐	☒	Estimates of effect sizes (e.g. Cohen's *d*, Pearson's *r*), indicating how they were calculated

Our web collection on statistics for biologists contains articles on many of the points above.

Software and code

Policy information about availability of computer code

Data collection	Data was retrieved from the public database as Microsoft Excel files.
Data analysis	Statistical analyses were conducted using JMP SAS Pro V.16 (SAS Institute, Cary, North Carolina). Sigmoidal regression, exponential limited growth regression, and Spearman rank correlation analyses were conducted in Sigma Plot Version 13.0 (Systat Software, San Jose, California, USA).

For manuscripts utilizing custom algorithms or software that are central to the research but not yet described in published literature, software must be made available to editors and reviewers. We strongly encourage code deposition in a community repository (e.g. GitHub). See the Nature Portfolio guidelines for submitting code & software for further information.

Data

Policy information about availability of data

All manuscripts must include a data availability statement. This statement should provide the following information, where applicable:

- Accession codes, unique identifiers, or web links for publicly available datasets
- A description of any restrictions on data availability
- For clinical datasets or third party data, please ensure that the statement adheres to our policy

Summary data are available in the Source Data supplementary file and all raw data are publicly available from the Coastal Protection and Restoration Authority

(CPRA) of Louisiana, via the Coastwide Reference Monitoring System program and can be retrieved from the Coastal Information Management System (CIMS) database (http://cims.coastal.louisiana.gov).

Research involving human participants, their data, or biological material

Policy information about studies with human participants or human data. See also policy information about sex, gender (identity/presentation), and sexual orientation and race, ethnicity and racism.

Reporting on sex and gender	N/A
Reporting on race, ethnicity, or other socially relevant groupings	N/A
Population characteristics	N/A
Recruitment	N/A
Ethics oversight	N/A

Note that full information on the approval of the study protocol must also be provided in the manuscript.

Field-specific reporting

Please select the one below that is the best fit for your research. If you are not sure, read the appropriate sections before making your selection.

☐ Life sciences ☐ Behavioural & social sciences ☒ Ecological, evolutionary & environmental sciences

For a reference copy of the document with all sections, see nature.com/documents/nr-reporting-summary-flat.pdf

Ecological, evolutionary & environmental sciences study design

All studies must disclose on these points even when the disclosure is negative.

Study description	Investigation of abiotic drivers of a vegetation dieback in the Mississippi River Delta
Research sample	16-year dataset of vegetation cover, marsh elevation, water levels, and salinity across 16 dieback monitoring stations and analysis of vegetation cover across 104 stations in marshes across the Louisiana coast
Sampling strategy	Dieback was observed in the Birdsfoot Delta. To test whether the dieback is reflected in the annual vegetation cover data at CRMS stations, we examined the 16-year timeseries of species cover data at CRMS stations in the BFD (n = 14), in all marshes where Phragmites was present (n = 74), and at a random subset of saline, brackish and freshwater marshes without Phragmites present (n = 30). Dieback was detected at sites with a relatively high coverage of Phragmites (> 20% for at least one year since 2007) and was concentrated in the BFD. Two sites outside of the BFD where Phragmites was dominant also experienced dieback (Fig. 2b). Subsequent analyses focused on comparing dieback and non-dieback sites and testing trends and relationships across dieback stations.
Data collection	All data were collected through the Louisiana Coastwide Reference Monitoring System (CRMS) Stations. The CRMS network is comprised of 391 monitoring stations across swamp, freshwater, intermediate, brackish, and saline wetlands with data collection beginning in 2006 and continuing to the present day. Each CRMS station is comprised of a 200-m x 200-m area where marsh elevation, marsh surface elevation change are measured, vegetation plots are located and soil cores are collected and other station measures (e.g., periodic plant biomass) are taken. At each station, continuous water level, salinity, and temperature data are recorded hourly from a nearby channel or bay. Rod Surface Elevation Tables (RSET) were established at each CRMS stations for measuring marsh elevation change over time.
Timing and spatial scale	Vegetation measurements at CRMS stations are collected during the summer, approximately June through August, each year. At each station, percent cover data is collected within ten permanent 2 m x 2 m plots along a 283 m transect using the Braun-Blanket cover class method. Water level and salinity data are collected hourly. Station elevations were surveyed in 2014 using an RTK-GPS. Marsh elevation change is collected twice per year (one RSET per station).
Data exclusions	Data from one CRMS station 0153 in the Birdfoot Delta was not included in the analysis as the marsh loss appeared to be through a different process of erosion (not dieback)
Reproducibility	Data analysis is reproducible
Randomization	Randomization was predetermined by the coastal monitoring design.
Blinding	Not applicable as cognizant subjects were not involved

Did the study involve field work? ☐ Yes ☒ No

Reporting for specific materials, systems and methods

We require information from authors about some types of materials, experimental systems and methods used in many studies. Here, indicate whether each material, system or method listed is relevant to your study. If you are not sure if a list item applies to your research, read the appropriate section before selecting a response.

Materials & experimental systems

n/a	Involved in the study
☒	☐ Antibodies
☒	☐ Eukaryotic cell lines
☒	☐ Palaeontology and archaeology
☒	☐ Animals and other organisms
☒	☐ Clinical data
☒	☐ Dual use research of concern
☒	☐ Plants

Methods

n/a	Involved in the study
☒	☐ ChIP-seq
☒	☐ Flow cytometry
☒	☐ MRI-based neuroimaging

Plants

Seed stocks	N/A
Novel plant genotypes	N/A
Authentication	N/A
