# Supplementary material for: Vegetation dieback in the Mississippi River Delta triggered by acute drought and chronic relative sea-level rise
Source: Nat Commun. 2024 Apr 25;15:3518. doi: 10.1038/s41467-024-47828-x (PMC11045820; doi:10.1038/s41467-024-47828-x)
Supplement: Supplementary file 3 — Description of Additional Supplementary Files [file 41467_2024_47828_MOESM3_ESM.pdf]

Description of Supplementary Files

Supplementary Data 1

Description: Annual data summary for total percent vegetation cover, *Phragmites australis* percent cover, seasonal mean and maximum salinity and marsh inundation at Coastwide Reference Monitoring System (CRMS) stations, Louisiana. Raw data for each CRMS station can be found at https://lacoast.gov/crms/#.
